# Revising “Nutritional Reference Values for Feeding at Evacuation Shelters” According to Nutrition Assistance by Public Health Dietitians Based on Past Major Natural Disasters in Japan: A Qualitative Study

**DOI:** 10.3390/ijerph181910063

**Published:** 2021-09-24

**Authors:** Noriko Sudo, Ikuko Shimada, Nobuyo Tsuboyama-Kasaoka, Keiichi Sato

**Affiliations:** 1 Natural Science Division, Faculty of Core Research, Ochanomizu University, Tokyo 112-8610, Japan; 2 Department of Nutrition, Faculty of Nutrition, University of Kochi, Kochi 781-8515, Japan; shimada@cc.u-kochi.ac.jp; 3 Section of Global Disaster Nutrition, National Institutes of Biomedical Innovation, Health and Nutrition, Tokyo 162-8636, Japan; ntsubo@nibiohn.go.jp; 4 School of Network and Information, Senshu University, Kawasaki 214-8580, Japan; satok@isc.senshu-u.ac.jp

**Keywords:** evacuation shelter, nutrition assistance, natural disaster, public health dietitian, Japan

## Abstract

It is important to provide nutritionally adequate food in shelters to maintain the health of evacuees. Since the Great East Japan Earthquake in 2011, Japan’s Ministry of Health, Labour and Welfare has released the “Nutritional Reference Values for Evacuation Shelters” (Reference Values) after every major natural disaster. There is clear evidence, however, that the Reference Values have only been used infrequently. This study aims to revise these guidelines to include the actual situation in the affected areas and the feasibility of the endeavor. This qualitative study uses group interviews with local government dietitians to propose revisions to Japan’s Reference Values. These revisions include the following: issuing Reference Values within 1 week of a disaster, showing one type of values for meal planning for each age group, showing the minimum values of vitamins, upgrading salt to basic components, creating three phases of nutrition (Day 1, Days 1–3, and After Day 4), stipulating food amounts rather than nutrient values, and creating a manual. Local government officials could use the Reference Values as guidelines for choosing food reserves, and dietitians could use them while formulating supplementary nutrition strategies for a model menu in preparation for disasters.

## 1. Introduction

Disaster relief and food assistance in refugee camps worldwide often use nutrition standards provided by international organizations, such as the World Health Organization (WHO) and the Sphere Project [1,2]. In Japan, however, after every cataclysm since the Great East Japan Earthquake in 2011, the government has provided national nutritional guidelines (“Nutritional Reference Values for Feeding at Evacuation Shelters”; hereafter referred to as “Reference Values”). Japan is perhaps the first country to have this kind of national standard. However, nutrition after disasters remains an ongoing concern in Japan. There is clear evidence that the Reference Values have been used only infrequently. After the Great East Japan Earthquake in 2011, only 13.8% of dietitians in the affected areas used the Reference Values [3]. According to a 2013 national survey, for instance, only 47.5% of municipalities reported knowledge regarding the Reference Values, of which only 6.5% reported using them [4]. Moreover, pneumonia resulting from poor nutrition accounts for nearly a quarter of all disaster-related deaths [5]; because evacuation shelter meals lack protein, the risk of aspiration pneumonia due to poor swallowing function is all too real [6,7]. Additionally, since the Great East Japan Earthquake, a succession of disasters involving earthquakes and torrential rain has occurred.

In emergency situations, food aid is difficult. Infants and young children are regarded as the most vulnerable, and many feeding guidelines for them during emergencies have been published [8,9,10]. However, as the global population is aging, we need to address the needs of older people as well. The United Nations High Commission for Refugees estimates that, on average, 10% of its caseload includes refugees aged >60 years [11]. Japan became an aging society (population aged 65 years and over >14%) in 1994, and the percentage of older citizens in 2020 was nearly 30%. Other developed countries will need nutritional requirements in emergencies for aged societies such as Japan in the near future.

Despite the ongoing problems surrounding nutrition after a disaster, there has been little research examining how the Reference Values have been used in actual disaster areas. The Fukushima Health Management Survey assessed dietary intake among evacuees; however, the postal survey was conducted 1 year after the Great East Japan Earthquake, and energy and nutrient intakes were not calculated [12,13].

The Dietary Reference Intakes (DRIs) for Japanese is revised every 5 years. Following the release of the 2020 edition of the DRIs, the Ministry of Health, Labour and Welfare (MHLW) is considering establishing new Reference Values. Because simply announcing the new Reference Values is not sufficient to ensure their widespread use, it is important to consider measures for revising and publicizing the Reference Values. Therefore, in this study, we examined policies adopted toward revising and circulating the Reference Values by conducting group interviews with local government dietitians who had experience in providing food to residents following natural disasters over the past decade. Our findings contribute to the processes of designing disaster food assistance in local governments by highlighting how to develop an evidence-based, locally adapted nutritional standard for nutritionally balanced and culturally tailored diets at evacuation shelters. Public health dietitians could also use our study to revise their nutritional practices during disasters for efficient and appropriate delivery of services.

## 2. Materials and Methods

### 2.1. Case Study Background

In Japan, municipalities provide primary support to disaster victims; their disaster management department establishes evacuation shelters and procures food. However, there are no dietitians, public health nurses, or other healthcare professionals in this department. Dietitians at regional prefectural public health centers support the municipalities in their jurisdiction. They procure missing items at the request of the municipalities and appropriately allocate them based on assessed needs. Additionally, they analyze dietary survey data collected from the shelters and coordinate with municipalities’ requests for external help. With technical help from prefectural dietitians, municipal dietitians, usually allocated at health departments, negotiate the nutrition packed into the bento meal boxes ordered and distributed at evacuation shelters by the disaster management department. For instance, dietitians might reduce the amount of fried foods and increase the amount of vegetables. These boxes are particularly important because survivors otherwise receive only stockpiled food and relief supplies, both of which mainly comprise carbohydrates, such as instant rice, sandwiches, and instant noodles [14].

In 2011, the MHLW provided guidelines for prefectures affected by the Great East Japan Earthquake by releasing Reference Values for meal planning at evacuation shelters in April and Reference Values for meal assessment in June (Table 1). The former indicates values of calories and four nutrients that ought to be packed into evacuation shelter meals, and the latter are used to assess the meals provided at the evacuation shelters on the basis of nutritional calculations. Since then, Reference Values for assessment have been issued to health authorities of the affected prefectures of each major natural disaster based on population composition of the affected prefecture and the Estimated Average Requirements (EAR) of the DRIs used at the time of the disaster.

### 2.2. Study Design

In this study, we selected three prefectures, each of which was affected by one of the three major disasters in the 2010s and was informed of the Reference Values. In the first prefecture, an earthquake occurred concomitant with a tsunami buffeting the coast. The second prefecture experienced an earthquake, and 4 years after the earthquake, it experienced torrential rains. There were torrential rains in the third prefecture. The first prefecture was chosen because it had the highest number of deaths, the second prefecture because it had the most damage from the earthquake, and the third prefecture because it had the highest number of cities flooded by the rains.

We conducted group interviews with public health dietitians in each of our chosen prefectures. We selected these dietitians by asking other dietitians currently working in the relevant prefectural governments to select those who, at the time of the disaster, had provided support to disaster victims as public health dietitians. Details of the selected dietitians are presented in Table 2.

### 2.3. Dates of Interviews

The interview for Prefecture Z was conducted via Zoom on 30 September 2020, with a total of five participants: two from the prefectural government, one from a prefecture-run health center, one from a city-run health center, and one city dietitian. The interview for Prefecture X was conducted via Zoom on 1 October 2020, with a total of five participants: two from the prefectural government, one from a prefecture-run health center, one city dietitian, and one town dietitian. The interview for Prefecture Y was conducted via Zoom on 19 November 2020, with a total of three participants: one from the prefectural government, one from a prefecture-run health center, and one from a city-run health center. An interview with one city dietitian and one town dietitian from Prefecture Y, who were unable to accommodate the original schedule, was conducted via Zoom on 4 December 2020.

### 2.4. Interview Content

Each structured interview was approximately 2 h long and was recorded on Zoom. The interview was then transcribed by a specialist agency, and the participants checked the transcriptions. The first author served as the interviewer, and all the authors attended all the interviews. For the questions that all participants could answer (for example, questions about experiences), all participants were requested to provide answers. For questions to which not all participants might have a reply (for example, questions about new ideas or opinions on revisions to the Reference Values and food aid practices), only those with specific responses were requested to speak. The questions were divided into two topics: those related to the revision of the Reference Values and those related to the revision of the “Simulator for calculating nutritional food stocks in preparation for large-scale disasters” published by the MHLW in 2020. This study reports only responses to the former. The questions related to the former part were as follows:

(1) How and for what were the Reference Values used?
(2) Were the Reference Values for meal planning and assessment used separately?
(3) Is there a need for Reference Values for stockpile planning that can be used during normal times?
(4) Are the types of nutrients covered and the values for them acceptable as they are?
(5) Are there any nutrients for which you think that new Reference Values are needed?
(6) Should the priority of nutrients be indicated for each phase?
(7) Are Reference Values for each age group needed?
(8) Is there a need to address chronic conditions other than hypertension?
(9) What is needed for the Reference Values to become more widely adopted?

### 2.5. Ethical Considerations

This study was reviewed and approved in accordance with the provisions of the Ethics Committee for Researches of Humanities and Sciences at Ochanomizu University (Notification No. 2020-35) and the institutional ethics committees of the National Institutes of Biomedical Innovation, Health and Nutrition (Notification No. KENEI-139). A letter of request, an interview guide, and a research cooperation consent form were mailed to participants in advance, addressing the individuals and the heads of their affiliated organizations. Participants then sent in their signed consent forms before the interview. To avoid identifying the local governments involved, the date and name of the disasters were not given. Because we considered it necessary to indicate the order of disaster occurrence to interpret the participants’ responses, we named the prefectures X, Y, and Z based on the order in which the disasters occurred.

## 3. Results

Overall, we found that the Reference Values were used by the dietitians as authorized evaluation indicators to persuade disaster management officials to improve the meals at shelters. However, dietitians did not separately use the two types of Reference Values (for meal planning and for nutritional assessment) and thought that only one set was enough. Conversely, the participants agreed with the need for new Reference Values for stockpile planning because stockpile planning would offer the opportunity for every local government to recognize the need for Reference Values. The Reference Values for each age group were hardly used because participants were often unaware of them; however, these values might have been used if the participants had known about them, suggesting the importance of publicity. Because of this lack of awareness, creating a manual demonstrating how to use the Reference Values in each phase and the preparations necessary to use them was considered essential.

### 3.1. Use of Reference Values

Other than one prefectural dietitian in Prefecture Z, whose assigned evacuation shelter closed down within a day, all participants used the Reference Values. A Prefecture X participant from the prefectural government noted that the Reference Values could be used to persuade disaster management officials to improve the meals (Table 3).

The separate use of Reference Values for meal planning and for assessment (Table 1), however, was more varied. Prefecture X, as an interviewee from the city government noted, could only use the Reference Values for meal planning. This was because those for assessment were not issued until 3 months after the earthquake and tsunami. Interviewees from this prefecture also noted that the values in both sets of Reference Values were quite similar, which is why individual sets of Reference Values were not necessary (Table 4). A Prefecture Y participant from the prefectural government recalled that they only used the Reference Values for assessment because they were not aware that there were two sets of values. In Prefecture Z, a city government interviewee stated that the Reference Values for meal planning were used for assessment, and interviewees again noted that only one set of Reference Values was necessary because the values in the two sets were similar to one another.

### 3.2. Need for Reference Values for Stockpile Planning

Participants from Prefecture X agreed on the need for Reference Values for stockpile planning because these values would then be shared with everyone; Reference Values issued after a disaster can only be viewed in the disaster areas (Table 5). Participants from Prefecture Y were more cautious; those from the city government likewise wanted Reference Values for stockpiling but were worried regarding whether local governments could meet the Reference Values with their stockpiling and hence recommended that minimum values be provided. The interviewee from the prefectural government stressed the need to specify food amounts rather than nutrient values and suggested that the Cabinet Office directly notify the department in charge of stockpiling. In Prefecture Z, the interviewee from the prefectural public health center again emphasized the importance of stipulating food amounts rather than nutrient values because administrative staff with no knowledge of nutrition purchased the stockpiles.

### 3.3. Types and Values of Nutrients Covered

When asked which of the five categories in Table 1 (for all people 1 year and older) and the four nutrients in Table 6 (for specific groups) were most helpful, participants from all the prefectures indicated that all categories in Table 1 were used. For Prefecture X, the prefectural government participant noted the difficulty in meeting the Reference Values, and the city government participant suggested that the values should be at a minimum and that not meeting them should be considered a serious liability. In Prefecture Y, the interviewees from the town and city governments observed the importance of simplifying the meal plan requirements for the bento meal box vendors. For instance, they suggested that a list of ingredients instead of nutrients would be helpful. Additionally, as with Prefecture X participants, the city government participant recommended minimum standards as more persuasive. The participants from the prefectural and city governments of Prefecture Z said that Table 1 was sufficient in the case of heavy rains where the length of evacuation and damages to commodity distribution were limited.

Interviewees also had recommendations regarding what should be included in the list of Reference Values. All prefectures agreed that sodium should be added to Table 1 (Table 7). The prefectural government interviewee in Prefecture X was reluctant to lengthen the list of Reference Values, although she recollected calculating additional values, including potassium and dietary fiber. Many of the participants from Prefecture Y likewise recommended adding dietary fiber, as did the participant from the prefectural health center in Prefecture Z. The Prefecture Z dietitians also suggested removing vitamin A.

### 3.4. Prioritizing of Nutrients

We asked whether nutrients should be prioritized according to the phases outlined in the 2011 MHLW report entitled “Lines of Thinking for Nutrition Improvement Measures at Evacuation Shelters” (Table 8), instead of the current blanket list of nutrients in Table 1 and Table 6. The participant from the prefectural public health center in Prefecture X recommended that the phases be shorter than 6 months because although the earthquake and tsunami disaster that hit the prefecture was said to be unprecedented in scale, there were only 1–2 months when food could not be purchased (Table 9). Prefecture Y’s prefectural dietitians indicated a need to be careful about the manner of presenting the information, fearing that administrative staff would say, “We have enough water and calories, so we do the meals later, right?” A participant from the city government of Prefecture Y suggested that showing the values as in Table 8 had its benefits and that there should be standard values for each phase. Just as with the participant from the prefectural public health center of Prefecture X, the prefectural public health center and city government interviewees from Prefecture Z recommended shortening the phases proposed in the MHLW 2011 report. They, in fact, specified reducing the months of the report to days or weeks in response to both health issues and complaints by residents about the meals. Additionally, the interviewee from the municipal public health center of Prefecture Z proposed that food examples be given for each phase of the diagram contained in the “Guidelines for Nutrition Assistance in the Event of a Large-Scale Disaster: What Local Government Officials Should Do,” published by the Japan Public Health Association [15], bearing in mind that non-dietitians will also look at the standard and suggest specific ingredients, such as “rice balls with a salmon filling” in place of “sweet buns,” could avoid the misconception that calorie intake alone is sufficient.

### 3.5. Reference Values for Each Age Group

In April 2011, the MHLW announced Reference Values (for those aged 1 year and older) in the left-hand side column of Table 1 and values for each age group in Table 10. When we asked whether Reference Values for each age group were necessary for the new Reference Values, all the interviewees from Prefecture X said that none of them used Table 10 and that it would not be necessary in the future. The participant from the city government, however, noted that she had not used Table 10 because she had not known about it (Table 11). Had she known about it, she might have used it and concluded that, following a disaster, it would be helpful to have as much information as possible Reference Values for each age group. While none of the participants from Prefecture Y used Table 10, they disagreed over whether they might have used it. The participant from the prefectural government stated that there were too many people in shelters to make sorting food according to age group viable; however, the town and city government interviewees believed that Reference Values for each age group would have helped with sorting food appropriately and quickly. The city government interviewee especially noted the difficulty of compiling meals for children. In Prefecture Z, the prefectural public health center participant remembered using Table 10 in a city, following the suggestion of the center, but one value (15–69 years old) was uniformly applied to all instead of the left-hand side column of Table 1; she could not remember the reason for which she had done so.

### 3.6. Adding Reference Values for Chronic Conditions

Of the current Reference Values, only sodium in Table 6 is set in relation to lifestyle-related diseases (the prevention of hypertension). When asked whether the new Reference Values should include nutrients to address chronic conditions other than hypertension, participants from all prefectures responded that they could not use them even if they existed and instead recommended using preexisting guidelines for each particular illness (Table 12). They offered a range of reasons for not being able to use Reference Values adjusted to particular chronic conditions. The city government and prefectural public health center participants from Prefecture X and the city government interviewee from Prefecture Z noted that it was not possible to tailor the bento meal boxes to individual patients. The city in Prefecture Z was able to provide special meals tailored to clinical conditions, diabetes, kidney disease, and dialysis patients only because of the small number of vulnerable people involved, along with assistance from the Association of Medical Doctors of Asia. The city government participant in Prefecture Y also noted that the similarity between disease guidelines for normal conditions and for disasters made it possible to use the guidelines for normal conditions when necessary after a disaster. The prefectural government participant in Prefecture Z feared that Reference Values for chronic conditions could lead to disparities among evacuation shelters as only those with external professional support could help the patients.

### 3.7. Increasing the Use of Reference Values

Many of the participants in this study learned about Reference Values only because of the disaster in their prefecture. To increase awareness of the Reference Values, the Prefecture X interviewees suggested including them in the work guidelines for administrative dietitians and in the DRIs, which are covered in the on-the-job training sessions held every 5 years when the DRIs are revised (Table 13). In the Prefecture Y interview, the prefectural government participant recommended creating a manual demonstrating how to use the Reference Values in each phase and the preparations necessary for using them; she had not used them herself because she could not envision how to put them into practice. In the Prefecture Z interview, the municipal government interviewee proposed producing a website that would contain all necessary information. However, the prefectural government interviewee added that people would probably not peruse the website unless it was absolutely necessary.

## 4. Discussion

This study was conducted to identify the challenges of food security responses after natural disasters in Japan. To the best of our knowledge, little research has been done on the utilization of nutritional guidelines on disaster sites. This study could lay the foundation for the development of guidelines in other countries.

### 4.1. Use of Reference Values

It has been indicated that scientific data collection and dietary surveys following the Great East Japan Earthquake were extremely limited [16]. Dietary surveys at shelters, however, were conducted by administrative dietitians following the three major natural disasters, although these data were rarely published as a scientific paper unless researchers were involved. As the interviewees were not fully aware of the usage of Reference Values, a dietary assessment manual for shelter meals needs to be compiled. Although the Iranian Ministry of Health has proposed formulations for emergency food basket and other supporting organizations have separate guidelines in this regard, the health and nutrition controlling guidelines of the stakeholders were not implemented in critical situations after natural disasters [17]. These facts imply that the need to provide not only the guidelines but also teaching material regarding how to use them.

### 4.2. Need for Reference Values for Stockpile Planning

Based on the nutrient reference values published by the Australian Government, Haug et al. [18] estimated that a food stockpile should provide an average energy intake of about 2150 kcal per person per day to avoid significant weight loss. However, this is near the average intake―men need a little more than women, while children and elderly need less. The new Reference Values will also be shown in normal times for stockpile planning and provided to accommodate differences in demographic characteristics among prefectures. Schofield and Mason [19] also pointed out that using one figure for setting the rations for all populations is inappropriate.

Some governments of developed countries have published guidelines for household food stockpiling that show which foods and in what quantities people are recommended to have [20,21]. However, food and nutrition guidelines for stockpiling by local governments have never been established.

### 4.3. Types and Values of Nutrients Covered

The interviewees noted the difficulty in meeting the Reference Values and suggested that the values should be at a minimum. Nutritional deficiencies have been widely observed in emergencies. Further, a significant rise in the incidence of malnutrition and all forms of vitamin and micronutrient deficiency was reported after natural or man-made disasters [22]. In particular, vitamin content in most rations is inadequate because they heavily rely on cereals [23]. Previous studies have reported that a beriberi epidemic occurred in a refugee camp [24], in which 55% of ration distribution provided less than half the riboflavin Recommended Dietary Allowance (RDA) [25]. Although it would be cheaper and require less manpower to rely on multivitamin tablets rather than main/side dishes to cover the requirements of vitamins, Japan’s nutrition policy considers supplementation as the last option and tries to first improve diets.

### 4.4. Prioritizing of Nutrients

**Figure 1:**
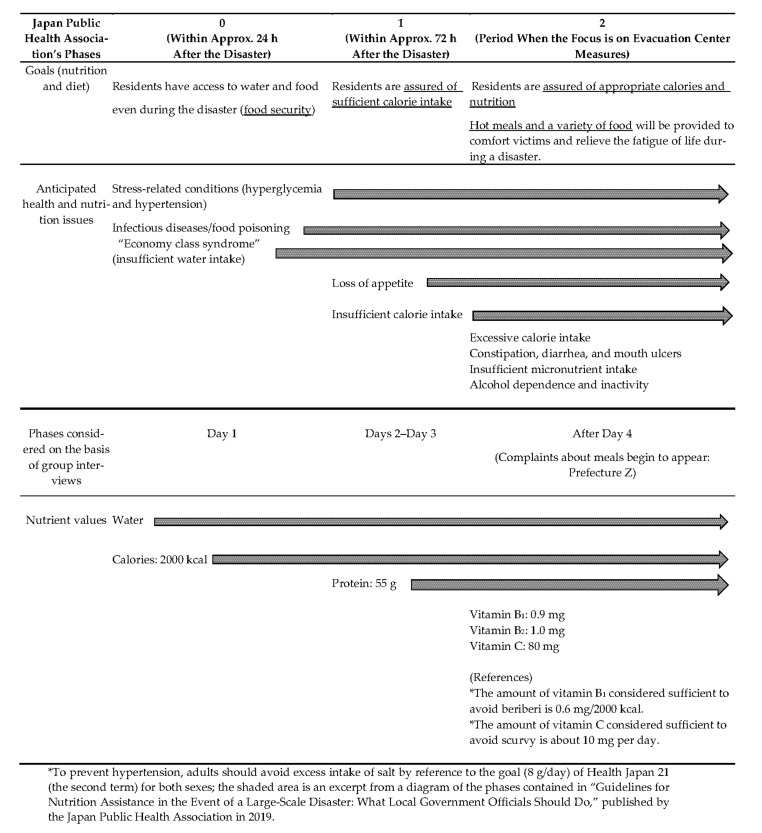
Proposed format for presenting the new nutritional reference values (white area).

The group interviews show that food scarcity following a disaster could be resolved in two months at the longest. The Reference Values should be given in shorter phases using phases 0–2 from the Japan Public Health Association guidelines [15], and specific examples of food items should be given as in Figure 1 and Table 14.

### 4.5. Reference Values for Each Age Group

Although a number of strategies, frameworks, policies, and guidance have been set out in relation to feeding infants and young children in emergencies, the needs of other age groups are seldom addressed [26]. Nor did our participants, except one, use Table 10. Given the variability and complexities of evacuees, however, it is evident that tailored nutrition assistance cannot be accomplished with a single template for those aged ≥1 year. In the group interviews, the participants presented some ideas of how Table 10 could be used had they known about it.

### 4.6. Adding Reference Values for Chronic Conditions

Many survivors of major disasters die shortly after the event, and hypertension is one of the most important risk factors for disaster-related issues [27]. Compared to nonevacuees, a greater increase in mean blood pressure was observed in evacuees after the Great East Japan Earthquake [28]. Increased exacerbations of cardiovascular diseases, including worsening control of hypertension and myocardial infarctions, with an associated increased risk of death, were observed after hurricane events associated with flooding [29]. It was reported that not only adults but also children reporting four or more traumatic experiences had marginally significant elevated levels of diastolic blood pressure [30]. Among the nine global targets for the prevention and control of noncommunicable diseases (NCDs) 2013–2020 [31], reduction of salt intake is only one dietary target and modifiable factor even in evacuation shelters. In the context of significance and implementability, hypertension might be the only chronic condition that needs to be addressed at shelters. All prefectures agreed that sodium should be added as one of the nutrients in Table 1, while they responded that they could not use other nutrients to address chronic conditions other than hypertension. Foods distributed during federal disaster relief response in Puerto Rico after Hurricane Maria contained a high amount of sodium [32]. High salt content in disaster food is a universally common characteristic because of food processing and long shelf life. In Caribbean regions, poor control of chronic NCDs was responsible for at least 30 percent of deaths after two recent hurricanes [33]. Management of NCDs in the setting of disasters in low- and middle-income countries would be the challenge.

### 4.7. Increasing the Use of Reference Values

There is currently an undergraduate education system for learning about Reference Values; however, it is limited and was put into practice after the participants in this study graduated from training colleges. In December 2010, the national examination standards for Registered Dietitian Nutritionists (RDNs) were revised to include “Health Crisis Management and Nutrition Assistance” as a sub-item of “Public Health Nutrition” and “Counter Measures for Accidents and Disasters” as a sub-item of “Food Service Management.” “Nutrition in Disasters” was then added as a sub-item of “Applied Nutrition” in February 2015. The textbooks for these specialized subjects for RDNs include tables of the Reference Values; hence, RDNs who have studied this curriculum will have learned about the Reference Values. However, the relevant sections of the textbooks vary widely in content and quantity [34], and a nationwide postal survey reported that nearly half of the students at Japan’s RDN training colleges did not understand them [35].

Provision of Reference Values for stockpile planning could get more people involved, including those without disaster experience. Creating a manual demonstrating the manner in which to use the Reference Values could contribute to detailed descriptions in the textbooks given that teachers with enough knowledge of disaster nutrition is limited [36].

## 5. Conclusions

This study has revealed potential ways of revising the Reference Values, circulating the revisions more widely, and examining the actual situation in Japan with respect to nutrition assistance during disasters. Based on the results of the aforementioned group interviews, a proposed policy for revising the Reference Values is outlined below.

- In the event of a disaster, receiving notice from the national government serves as an impetus for disaster management staff to make improvements to meals; hence, the Reference Values tailored to age–number distribution of people in affected prefectures should be issued in writing within 1 week of the disaster.
- Only one type of value for meal planning should be issued.
- A manual should be created that explains different features in meal assessment at shelters where individuals’ intakes are difficult to measure. For assessment, the amount of provision should be compared with EAR and RDA of DRIs.
- The Reference Values should be used under normal conditions for stockpiling. In addition to a basic standards for the whole Japanese population, an Excel Sheet that shows amounts of calories and nutrients, calculated by weighing the DRI values by the gender and age ranges of the city/town/village, should be presented to reflect differences in demographics so that each municipality can find a precise scheme.
- To prevent nutritional deficiencies, current Reference Values for meal planning are RDA-based. For vitamins B_1_, B_2_, and C, however, they should be EAR-based because even EARs are set beyond the minimum necessary intake that prevents deficiencies.
- A Reference Value for sodium should be added to those for calories, protein, and vitamins B_1_, B_2_, and C in Table 1. The nutrients for specific groups in Table 6 are unnecessary. Other nutrients deemed necessary in specific situations could be calculated using the DRI values.
- The Reference Values should be given in shorter phases than the monthly phases in Table 8. Phases 0–2 from the Japan Public Health Association guidelines should be used, and specific examples of food items should be given (Figure 1 and Table 14).
- If the Reference Values by age group are to be used as a meal plan, it would be for individualized support. The combination of relief supplies that can meet these nutritional requirements should be shown for each age group (for one meal and for 1 day), and the characteristics of particular life stages, such as infancy and old age, should be considered. Table 10 is a reference for assessing the sufficiency of nutrients for children.
- There is no need to address particular medical conditions other than hypertension.
- To circulate information among dietitians with no personal experience with disasters or work in disaster support and to enhance undergraduate education, the use of Reference Values should be explained in the manual.

Under normal conditions, local government officials could use Reference Values as the guideline for choosing food reserves. Dietitians could also use them while formulating supplementary nutrition strategies for creating a model menu in preparation for disasters.

In addition to the revisions of the Reference Values, the group interviews showed the importance of facilitating cooperation between different departments within the local government and among different professionals, such as dietitians, non-dietitians, and bento vendors. In future studies, we intend to conduct another round of group interviews with the same public health dietitians to confirm whether we correctly reflected their opinions in the current revisions. Furthermore, we will conduct new group interviews with disaster management personnel who are in charge of purchasing stockpile foods to check if the new Reference Values and their manual are understandable.

## Figures and Tables

**Table 1:** Nutritional Reference Values at evacuation shelters (per day and per person aged 1 year and above).

	For Meal Planning (Based on RDA) ^a^	For Dietary Assessment (Based on EAR) ^b^
Energy (kcal)	2000	1800–2200
Protein (g)	55	≥55
Vitamin B_1_ (mg)	1.1	≥0.9
Vitamin B_2_ (mg)	1.2	≥1.0
Vitamin C (mg)	100	≥80
Date of release	21 April 2011	14 June 2011

Note. RDA, Recommended Dietary Allowance. EAR, Estimated Average Requirement. ^a^ Japanese Ministry of Health, Labour and Welfare (2011). Nutritional Reference Values to Be Used as Near-term Targets for the Planning and Assessment of Meal Provision in Evacuation Shelters. ^b^ Japanese Ministry of Health, Labour and Welfare (2011). Implementation of Appropriate Nutrition Management in Meal Provision at Evacuation Shelters.

**Table 2:** Number and categorization of local government dietitians who participated in group interviews.

	Earthquake/Tsunami (Prefecture X)	Earthquake (Prefecture Y)	Heavy Rain (Prefecture Z)	Total
Prefectural Government	2	1	2	5
Prefecture-run Public Health Center	1	1	1	3
City-run Public Health Center	0	1	1	2
City Government	1	1	1	3
Town Government	1	1	0	2
Total	5	5	5	15

**Table 3:** How and for what were the Reference Values used? (Q1).

Prefecture X (Earthquake and Tsunami)
We evaluated the results of the dietary survey using DRIs; however, one month after the disaster, the MHLW provided the Reference Values to use as standards during disasters. This made it easier to persuade those in charge of supplies to prioritize improving the meals (Prefectural Government).
Prefecture Y (Earthquake) (the statements below refer to usage during the second torrential rain disaster)
As this was the second disaster, the public health center took the lead in conducting a dietary survey alongside the prefectural government, establishing an assessment process using the Reference Values (Prefectural Government). A few days after the disaster, a dietary survey was conducted at two evacuation shelters where catered meal boxes were distributed (City-run Public Health Center).
Prefecture Z (Torrential Rains)
To get improvements made to the meals, we needed to make proposals based on nutritional calculations. A document titled “Proposals for the Implementation of Appropriate Nutritional Management in the Provision of Meals at Evacuation Shelters” was issued in the name of the director of the prefectural health and welfare department, addressed to the heads of public health center in a core city in the prefecture, and this included the results of nutritional calculations. The Reference Values were used for this assessment (Prefectural Government). From about the third day, three boxed meals were provided each day. Just by looking at them, I could tell that they were lacking in vegetables, but I confirmed this in numerical terms by using the Reference Values (City Government).

**Table 4:** Were the Reference Values for meal planning and assessment used separately? (Q2).

Prefecture X (Earthquake and Tsunami)
As the values (for planning and assessment) do not differ much, I don’t think we need to be so strict and can just use one rounded number (City Government).
Prefecture Y (Earthquake)
We weren’t really aware (that there were two types of Reference Values) (Prefectural Government). This group interview made us aware (of the two types of Reference Values) (Prefecture-run Public Health Center, City Government).
Prefecture Z (Torrential Rains)
Complaints about the meals started to come up quite often from day 3 or 4. Once there was a significant number of people asking for a change to the meals, the upper management of the municipalities would take action; thus, it would have been helpful if the word had got to them earlier (in fact, notice of the assessment values was given about a month later). Just one would be enough (City Government).

**Table 5:** Is there a need for Reference Values for stockpile planning that can be used during normal times? (Q3).

Prefecture X (Earthquake and Tsunami)
We might not have seen (the current Reference Values) if the disaster hadn’t happened, so if they are for stockpiling purposes, they should be shared with everyone (Town Government).
Prefecture Y (Earthquake)
As stockpiling is done by a completely different department, it would be useful if Reference Values were presented under the explicit name of “stockpiling plan” and if the officials in charge of disaster management could be given notice of stockpiling through specific examples. In particular, it is important to use specific foods rather than nutrient values. When the people in charge of nutrition talk about it, it doesn’t go anywhere, but if the Cabinet Office directly notifies them, it may work. As such, if the Reference Values are also directly communicated to the disaster management officials from the perspective of evacuation shelter management, it may lead to them being applied when stockpiling is conducted. Reference Values will serve as a basis for stockpile planning; thus, I think they would make it easier for public health officials to say that this is the amount needed so that disaster prevention officials to think about it. However, in reality, I don’t think it will be possible for local governments to stockpile while also meeting the Reference Values. I think it’s good to have Reference Values as an ideal, but I also think it would help the actual people in charge if the Reference Values were feasible, showing the very minimum necessary (City Government).
Prefecture Z (Torrential Rains)
You can stockpile dried foods to meet vitamin and calcium needs, but they are just a single item and need to be cooked before serving. I was wondering if there is any point in making up numbers for nutrients that can’t be translated into actual foods (Prefecture-run Public Health Center).

**Table 6:** Nutritional Reference Values for meal assessment—nutrients requiring consideration for specific groups.

Purpose	Nutrients	Considerations Relating to Specific Characteristics (Excerpt)
Avoiding insufficient nutrient intake	Calcium	For bone mass accumulation, especially in 6–14-year-olds, 600 mg/day is recommended, with a varied food intake
	Vitamin A	To prevent growth impairment, intake should be no less than 300 μg RAE/day, especially for children aged 1–5 years, and attention should be paid to the intake of main and side dishes
	Iron	Menstruating persons with a history of anemia should undergo professional evaluation by a physician or dietitian
Primary prevention of lifestyle-related diseases	Sodium (salt)	To prevent hypertension, avoid excessive intake in adults, referring to the target amount

Note. RAE, Retinol Activity Equivalent. Matters of concern regarding excessive or inadequate nutrient intake after 3 months; Japanese Ministry of Health, Labour and Welfare (2011). Implementation of Appropriate Nutrition Management in Meal Provision at Evacuation Shelters.

**Table 7:** Types and values of nutrients covered. (Q4 and Q5).

Prefecture X (Earthquake and Tsunami)
After a considerable amount of time had passed, I managed to meet the Reference Values for assessment (right column of Table 1). The difficulty is with sodium. Evacuation shelters often provide soups and processed foods; thus, trying to meet the nutritional requirements will always result in more sodium (Prefectural Government). If the nutrient values are considered in isolation, it can lead to the perception that vitamin supplements are sufficient, which is far from actual meals. Eating something warm and feeling at ease are also important elements of food, so I think it would be better to set the values at levels that are not too demanding. Reference Values are used to assess whether the food provided by the local government as a government service to evacuees is sufficient, and if it is insufficient, it may be seen as a serious liability issue, so a basic minimum value is preferable. Additionally, as these are the figures that we dietitians will use as a basis for appealing to our superiors and the disaster response headquarters, it is better to use minimum values that are easily achievable. When I strongly suggested to the administrative staff that we provide nutritious meals at the evacuation shelters, they said that no one thinks about nutrition that much in their daily lives, so why should we go to such lengths (during disasters), which is why I think it is definitely important to have a minimum baseline. We also faced the dilemma of the nutritional disparity between victims in evacuation shelters and those staying at home, so we would appreciate being given realistic values rather than the ideal (City Government).
Prefecture Y (Earthquake)
We calculated all nutrients in Table 1 besides fat, carbohydrates, sodium, and equivalent salt amounts. This has to be something that the administrative staff knows about, rather than the less well-known nutrients; otherwise, they won’t remember it. I focused on the nutrients found on nutrition facts in food labels. Supplements are delivered to the evacuation shelters quite often, so we can make up for any shortages with those. Foods served in shelters are quite high in salt. For now, the first question is whether it is balanced, and the ones after that, like vitamins, need to be explained. We can’t explain directly to our more senior supervisors, so we chose the nutrients that can be easily understood at a glance (City-Run Public Health Center). I used only Table 1 and did not look at Table 6 much. For vitamin B_1_, if you negotiate with the bento vendor saying, “Please try to use what you can from this food list because it is contained in these ingredients,” it will be easier to select this and that for the day’s menu, like a puzzle (Town Government). In our city, the local restaurant association has a bento section, and the disaster management department requested a weekly meal plan prepared by dietitians dispatched from another prefecture. We provided the vendors with a meal plan with only calories, protein, fat, and salt listed on it. If the Reference Values were translated into actual ingredients, I think it would be easier for the suppliers to use (City Government).
Prefecture Z (Torrential Rains)
For meal assessment, I used dietary sodium and fiber besides Table 1. I added the former two because when I saw the bentos, I was concerned about the amount of salt and the lack of vegetables (Prefecture-run Public Health Center). The new Reference Values should include all of Table 1 plus calcium, iron, and sodium from Table 6. If you are going to live in an evacuation shelter for more than 3 months, it would be better to have various nutrients, like those in Table 6. However, because these torrential rains did not last that long and the medical response was not that extensive, I felt that Table 1 was already sufficient. Much depends on the type and scale of the disaster (City Government).

**Table 8:** Lines of thinking for nutrition improvement measures at evacuation shelters (excerpt).

Phases	Approaches to Improving Nutrition
Within 1 month	Ensure water and calorie intake
1–3 months	Ensure intake of minimum requirements and prioritize supplementation of nutrients that are stored in the body in small amounts (calories, protein, vitamin B_1_, vitamin B_2_, and vitamin C) Ensure frequency and quantity of meals Consider the use of nutrient-added foods (e.g., fortified rice)
3–6 months	Insufficient nutrient intake is considered in relation to specific characteristics (calcium, vitamin A, and iron) Consider excessive intake of calories and certain nutrients Ensure meals comprise a staple food, a main dish, and side dishes
6 months onwards	Consider primary prevention of lifestyle-related diseases Ensure meals that meet each individual’s health needs, comprising a staple food, a main dish, and side dishes

Note. Japanese Ministry of Health, Labour and Welfare (2011). Lines of Thinking for Nutrition Improvement Measures at Evacuation Shelters.

**Table 9:** Should the priority of nutrients be indicated for each phase? (Q6).

Prefecture X (Earthquake and Tsunami)
It might be better to have a list of priorities for shorter spans of time than 6 months. I am doubtful whether it is useful to think in such a long timeframe. Two or three months after the disaster, we were being told things like, “If the food is just sweet buns like this, everyone will develop diabetes. You’re going to give everyone diabetes.” However, now I look at Table 8 and think that perhaps I didn’t have to worry about it until six months had passed. The period during which people can’t eat isn’t that long. After the earthquake and tsunami, there was a period of one or two months when people could not buy anything; however, during other disasters, stores were open next to evacuation shelters, and even during typhoons, there were places where people could buy things immediately. Conversely, if it is a long period of time like in Table 8, I sometimes wonder how much support municipalities should provide. I think the timeframe should be a little shorter (Prefecture-run Public Health Center).
Prefecture Y (Earthquake)
“Ensure calorie intake” in the “within one month” category refers only to nutrients and does not include the perspective that eating a meal can calm people down or that being provided with food as usual will restore their mental balance. Even if you have ensured sufficient caloric intake, there’s the question that is it enough to eat rice balls and bread every day, so it’s important to present a different perspective. There will be people who think that “as long we can ensure caloric intake for now, that’s all that’s needed, and we’ve been able to procure this much, so it’s fine.” If we don’t think about how we use and present this information, it will lead to misunderstandings (Prefectural Government). It would be nice to have standard values for each phase. I think it’s good to have them for each phase because that way you can see that the perspective changes when it comes to the long term. It would be better if the values were included in Table 8 (City Government).
Prefecture Z (Torrential Rains)
During the “within one month” period, we were working to ensure that the nutrients in Table 1 were met. It would be helpful to have targets for vegetable intake and intake by food groups (City-run Public Health Center). After 3 or 4 days, the residents complained about every aspects of meals, including times per day. “Within one month” in Table 8 could be broken down more finely, such as “within 3 days” and “4 days to 1 week” (City Government).

**Table 10:** Nutritional Reference Values for planning by age group.

Reference	Per Person Per Day			
	Infants (Aged 1–5 Years)	Growth Period I (Aged 6–14 Years)	Growth Period II and Adults (Aged 15–69 Years)	Elderly People (Aged 70 Years and above)
Energy (kcal)	1200	1900	2100	1800
Protein (g)	25	45	55	55
Vitamin B_1_ (mg)	0.6	1.0	1.1	0.9
Vitamin B_2_ (mg)	0.7	1.1	1.3	1.1
Vitamin C (mg)	45	80	100	100

Note. Japanese Ministry of Health, Labour and Welfare (2011). Nutritional Reference Values to Be Used as Near-term Targets for the Planning and Assessment of Meal Provision in Evacuation Shelters.

**Table 11:** Are Reference Values for each age group needed? (Q7).

Prefecture X (Earthquake and Tsunami)
Reference Values are referred to as “Reference” here (the upper left column of Table 10), so if it is something that can be used for that purpose, there isn’t a problem in having it there. I didn’t know about Table 10, but if I had, I might have looked at it. You can’t predict what you will want to know, so it’s worth having a reference to look at (City Government).
Prefecture Y (Earthquake)
Because there are so many people mixed in the evacuation shelters, this would not be usable even if we had it. First, there’s no situation where you have an evacuation shelter full of only old people. You can’t divide people up into neat groups in these circumstances. Because the aging rate in our prefecture is quite high, I think it would be good to have a single category of “elderly people” from now on. However, if you ask how much of a difference there will be compared to the left column in Table 1, I think the actual meals provided will only require slight adjustments. In fact, it is the elderly who use the shelters for three meals a day. Those who are healthy and able to go to work are asked to take care of their nutrition themselves; however, when we think about those who can only eat at the shelters, I think it would be good to consider Reference Values for use with elderly people too (Prefectural Government). Although these subdivisions make it possible to conduct more detailed assessments, dividing people into these groups and performing the assessments isn’t very realistic. It would be easier to use a single value that considers the composition of the population in advance rather than having many values as in Table 10. Having to pick this and that for each of the four groups every time would be very difficult as well. For those who had difficulty in swallowing and needed individual attention, we handed out rice porridge and soft food that had been delivered as relief supplies. At that time, the amount of food to be given out was divided into single meal or 3-day portions by the dietitians who were assigned to us based on its approximate calorie count. In such situations, I think that these kinds of numbers would help in sorting out the food quickly. By comparing the results of the assessment, it would be easy to confirm that, say, people in Growth Period II and Adult category are not getting enough nutrients. It would then be possible to add some additional foods and distribute them to those people. However, I’m not sure how much we could do on the ground (Town Government). When providing meals at evacuation shelters, I don’t think it’s possible to do so separately for each age group. Given that we don’t, there’s no need for this. Although there weren’t many consultations regarding children, I thought that it would be worth having some standard values when providing individual support. It was a matter of choosing suitable foods from the special diets in the relief supplies and giving them to people as well as providing ideas on how to eat them. Thus, we didn’t cook for people individually, and I don’t think it would have been possible. We didn’t even think about the amounts of nutrients, we just gathered up what we thought would be easy to eat and gave it to them. We could use Table 10 to say that if we get a certain amount of food, then that should be enough for children (City Government).
Prefecture Z (Torrential Rains)
Reference Values for each age group would be difficult to use even if we had them. We can’t order special bentos for the elderly who are in the evacuation shelter all day just for lunch. We can’t do that kind of fine-tuning, so even if we had these values, it wouldn’t really... (Prefectural Government). The one I presented to the city had 2100 kcal, so I think I did use this. I applied a uniform 2100 kcal for the 15–69 age group to everyone. I don’t remember why I preferred this one to the 2000 kcal in Table 1 (Prefecture-run Public Health Center).

**Table 12:** Is there a need to address chronic conditions other than hypertension? (Q8).

Prefecture X (Earthquake and Tsunami)
We need to be alert to prevent obesity and diabetes. Volunteers set up food stalls around the evacuation shelter, like at a festival, and a large amount of sweets also arrive as relief supplies. It would be nice if there was something that could put a stop to this, but it may be difficult to indicate that in terms of Reference Values. However, in terms of medical conditions, I think that a system provides individual nutritional guidance to those who require special consideration is more important than Reference Values (Prefectural Government). In evacuation shelters, it is not possible to provide bentos tailored to each patient, so they end up being the same. Perhaps we can give considerations on an individual level and speak with those with special requirements. For example, they could avoid using the soy sauce that comes with the bento or leave some of the food if there is too much (Prefecture-run Public Health Center). If there are too many detailed standards, it will be difficult to separate people with a particular condition from everyone else for each meal (City).
Prefecture Y (Earthquake)
Even if Reference Values are given, I think it would be very difficult to use them for conditions other than hypertension. There aren’t many people who self-report that they have high blood pressure (Prefectural Government). They would be very difficult to apply in an evacuation shelter. The lack of guidelines has never caused me any difficulties in dealing with a sick person before (Prefecture-run Public Health Center). There was a situation where people with illnesses were bringing their stockpiled special food for their conditions and eating it themselves at evacuation shelters (City-run Public Health Center). As the values do not differ greatly between disaster conditions and normal conditions, the disease guidelines can be used (City Government).
Prefecture Z (Torrential Rains)
If special meals were to be served in the prefecture’s core cities, all evacuation shelters would have to take the same measures, which was not possible in some cases. The bento suppliers will have to deal with this on a case-by-case basis, so if there are a lot of special meals, they will be able to handle it; however, if there are only one or two in a given evacuation shelter, they probably won’t be able to cope. So, I wonder if these extra values are really needed (Prefectural Government) It will be difficult to coordinate bentos to accommodate different meals for different health conditions. I think it will be more of an individualized approach (Prefecture-run Public Health Center). We served special meals to some people with diabetes, kidney disease, or those on dialysis. The Association of Medical Doctors of Asia doctor also gave us some instructions, and we served food based on them twice a day; however, in general, we could only give blanket instructions to the bento suppliers, such as to lower the salt content to a certain level. I think it would be quite difficult to change the calorie values for each person individually (City Government).

**Table 13:** What is needed for the Reference Values to become more widely adopted? (Q9).

Prefecture X (Earthquake and Tsunami)
The unaffected areas did not know about the Reference Values at all. It would be good to include them in something like the work guidelines for public health dietitians or in some section of the DRIs (Prefectural Government). If they are in the DRIs, many people will participate in the relevant training sessions, so it could spread quite a bit from there (Prefecture-Run Public Health Center).
Prefecture Y (Earthquake)
I couldn’t really imagine how to apply them in practice. If I had a concrete idea of how to use the Reference Values, for example, by conducting an assessment after about half a month and presenting requests for improvement to the bento suppliers, I might have been able to use them right away. In the end, we were able to notify all the bento vendors and ask them to make improvements, but this involved a lot of hard work while doing other things; thus, I later thought that if we had drawn up some sort of template, we could have saved ourselves a lot of trouble (Prefectural Government). It was only after my own area was hit by a disaster that I actually used them. Unless there is a situation where we can use them, it just stays as knowledge, and we can’t put them into practice (Prefecture-run Public Health Center).
Prefecture Z (Torrential Rains)
You wouldn’t think about them unless you actually experience a disaster (Prefectural Government). If there were a national government website where you can find a list of all the things you need to know about disasters or food, then people could be made aware of it (City Government).

**Table 14:** Examples of foods for meeting the new Reference Values.

Phases	Phase 0: Day 1	Phase 1: Day 2–Day 3	Phase 2: After Day 4
**Nutrients to be considered and their Reference Values**	**Water** **Calories: 2000 kcal**	**Water** **Calories: 2000 kcal** **Protein: 55 g**	**Water** **Calories: 2000 kcal** **Protein: 55 g** **Vitamin B_1_: 0.9 mg** **Vitamin B_2_: 1.0 mg** **Vitamin C: 80 mg**
**Examples of foods for meeting the above Reference Values**	**Day 1** **Breakfast:** Canned bread 100 g Water **Lunch:** Porridge 250 g Hardtack 100 g Water **Dinner:** Pregelatinized rice 100 g Rice cookies 100 g Water **Calories: 1820 kcal**	**Day 2** **Breakfast:** Udon in a cup (noodles made from wheat flour) 75 g Canned mandarin orange 50 g Water **Lunch:** Pregelatinized rice 100 g Vegetable mix juice 200 mL Mackerel simmered in miso sauce 90g **Snack:** Rice cookies 70 g **Dinner:** Pregelatinized rice 100 g Canned grilled chicken 75 g Canned boiled soybeans 50 g marinated with dried salty kelp 5 g Water **Calories: 1924 kcal** **Protein: 55 g**	**Day 4** **Breakfast:** Bread roll 30 g * 2 rolls Orange juice 150 mL Fish sausage 90 g Corn cream soup 150 g **Lunch:** Pregelatinized rice 100 g Chicken curry (retort) 200 g 1 banana Green tea 200 mL **Snack:** Dried sardine with almond 30 g **Dinner:** Boiled rice with barley 250 g Canned tuna and asparagus marinated with mayonnaise 70 g Miso soup with soy milk (boiled bamboo shoots 20 g, potatoes 40 g) 1 bowl Green tea 200 mL **Calories: 1975 kcal** **Protein: 60 g** **Vitamin B_1_: 0.8 mg** **Vitamin B_2_: 1.5 mg** **Vitamin C: 126 mg** **Salt: 8.8 g**
		**Day 3** **Breakfast:** Porridge 250 g Canned barbecued Brevoort 50 g Biscuits 30 g Water **Lunch:** Canned bread 100 g Vegetable mixed juice 200 mL Canned seasoned beef 50 g **Snack:** Azuki-bean jelly 50 g **Dinner:** Pregelatinized rice 100 g Green tea 200 mL Chicken stew 200 g Canned sardines in tomato sauce 50 g Canned mandarin orange 50 g **Calories: 1787 kcal** **Protein: 58 g**	**Day 5** **Breakfast:** 1 sliced bread 60 g Peanut butter 13 g 1 egg 55 g Yogurt 80 g Grapefruit juice 150 mL **Lunch:** Fortified rice 200 g Beef stew (retort) 200 g Canned boiled soybeans 50 g Canned pineapple 50 g Green tea 200 mL **Dinner:** 2 rice balls with laver 250 g Canned seasoned sardines 50 g 1 tomato Somen noodles in soup with breast chicken (canned, boiled), dried white radish, brown seaweed, and dried mushrooms 180 g Green tea 200 mL **Calories: 1722 kcal** **Protein: 60 g** **Vitamin B_1_: 1.0 mg** **Vitamin B_2_: 1.3 mg** **Vitamin C: 146 mg** **Salt: 5.5 g**
			**Day 6** **Breakfast:** Bread roll 30 g * 2 rolls Corn flakes 40 g Milk 100 g 2 slices of ham 20 g 1 cucumber 100 g 1 egg 55 g Apple juice 150 mL **Lunch:** Boiled rice with barley 250 g Chicken stew 200 g (with canned boiled mushrooms 30 g) Cheese 18 g 1 Mandarin orange 80 g Green tea 200 mL **Dinner:** Fortified rice 250 g Clams boiled in soy sauce 10 g Marinated canned tuna in oil, broccolis, and cherry tomatoes 200 g Green tea 200 mL **Calories: 1872 kcal** **Protein: 61 g** **Vitamin B_1_: 1.2 mg** **Vitamin B_2_: 1.4 mg** **Vitamin C: 138 mg** **Salt: 6.8** g
			**Day 7** **Breakfast:** 2 rice balls with dried bonito 250 g Fried Spam and bamboo shoots 80 g Vegetable mixed juice 200 mL **Lunch:** Boiled rice with barley 250 g Kenchin soup with quail eggs and tofu 250 g Canned peach 50 g Green tea 200 mL **Dinner:** Fortified rice 250 g Tomato soup with mackerel 300 g Green tea 200 mL **Calories: 1820 kcal** **Protein: 60 g** **Vitamin B_1_: 1.1 mg** **Vitamin B_2_: 1.2 mg** **Vitamin C: 115 mg** **Salt: 3.1 g**

* To prevent hypertension, adults should avoid excess intake of salt by reference to the goal (8 g/day) of Health Japan 21 (the second term).
